# Bulimia symptoms and anger and aggression among adolescents

**DOI:** 10.1186/s12889-023-15664-1

**Published:** 2023-05-05

**Authors:** Roman Koposov, Andrew Stickley, Denis Sukhodolsky, Vladislav Ruchkin

**Affiliations:** 1 Regional Centre for Child and Youth Mental Health and Child Welfare, Faculty of Health Sciences, UiT The Arctic University of Norway, Tromsø, Norway; 2 Sechenov First Moscow State Medical University, Moscow, Russia; 3 Department of Preventive Intervention for Psychiatric Disorders, National Institute of Mental Health, National Center of Neurology and Psychiatry, Kodaira, Tokyo Japan; 4 Stockholm Center for Health and Social Change (SCOHOST), Södertörn University, Huddinge, Sweden; 5 Child and Adolescent Psychiatry Unit, Department of Neuroscience, Uppsala University, Uppsala, S-751 85 Sweden; 6 Child Study Center, Yale University School of Medicine, New Haven, CT USA; 7 Sala Forensic Psychiatric Clinic, Sala, Sweden

**Keywords:** Bulimia, Anger, Aggression, Rumination, Adolescents, Gender

## Abstract

**Background:**

Previous research has indicated that anger and aggression may be elevated in adolescents with a bulimia nervosa (BN) diagnosis. However, as yet, little is known about whether bulimia symptoms are linked to anger and aggression in adolescents in the general population. To address this deficit this study aimed to explore the associations between a clinical level of bulimia symptoms (CLBS) and anger, anger rumination and aggression in community-based adolescents, and determine whether gender is important in this context.

**Methods:**

This study was conducted on a representative sample of youth from northwestern Russia *(n* = 2613, age 13–17 years old, 59.5% female) using self-report scales. A proxy variable for a CLBS was created using the Eating Disorder Diagnostic Scale. Aggression, anger and anger rumination were assessed by the Trait Anger Scale of the State Trait Anger Expression Inventory, the Anger Rumination Scale, and scales created to assess physically and verbally aggressive behavior. Multivariate analysis of covariance was used to examine the associations between the study variables.

**Results:**

A CLBS was more prevalent in girls than in boys (13.4% vs. 3.5%). The association with anger and aggression was stronger in both genders with a CLBS, compared to those adolescents without a CLBS. In the CLBS group, boys as compared to girls scored higher on verbal and physical aggression, anger rumination and social aggression. In both the CLBS and Non-CLBS groups higher anger and aggression scores were associated with increasing age.

**Conclusions:**

Findings suggest that aggression and anger rumination are elevated in adolescents with BN symptoms, and that the associations between anger, aggression and BN symptoms may be stronger in boys. As previous research has indicated that the presence of aggressive behaviors may affect the prognosis of BN and complicate management of the disorder, clinician screening for these behaviors in adolescents with BN symptoms may facilitate the provision of more effective treatment, especially among boys.

## Background

Bulimia nervosa (BN) is a severe and complex psychiatric disorder characterized by recurrent episodes of eating large amounts of food with an associated loss of control, that are followed by weight-related compensatory behaviors (including self-induced vomiting, misuse of laxatives or diuretics, fasting, or excessive exercise) and an overestimation of one’s own body size and weight [1]. BN symptoms are difficult to treat [2] and can have a significant negative impact on both physical and psychological development [3, 4]. Community-based research has indicated that while the lifetime prevalence of a BN diagnosis is relatively low among adolescents, ranging between 0.4% and 1.6% [5–8], the prevalence of compensatory behaviors associated with subthreshold bulimic symptoms may be relatively high, e.g. as high as 23.6% for weight loss and 23.1% for food restriction [9], producing medical and psychological complications comparable to those of the full-threshold disorder [10, 11]. Although BN symptoms are more common in females [12], their prevalence in males tends to be underestimated [13] and there is a relative shortage of information on eating problems in boys from the general population [14, 15].

Previous research has indicated that individuals with BN often have problems with emotional regulation (e.g. negative affectivity [16], and impulse control [17]), which may potentially increase the risk for comorbid conditions/behaviors with similar features, such as affective and anxiety disorders [18], suicidal behavior [19], binge drinking [20, 21] and obsessive–compulsive disorders [22]. In addition, impulsivity and negative affect in individuals with eating problems, and particularly with BN, have been linked to aggression, both in adults [23] and adolescents [24–26], which may further interfere with their functioning and impact treatment outcomes [23, 27]. As suggested by Miotto et al., [24], the development of disordered eating, especially in bulimia spectrum disorders, might be potentiated by problems in dealing with aggressive behavior and hence, disordered eating may serve as a compensatory mechanism which aims to soften the negative emotional burden associated with aggression. While frequently observed in clinical samples, the association between bulimic symptoms and aggression in adolescents from the general population has been less explored [25].

Aggression is a complex social behavior with many causes and manifestations, aimed at harming others physically or psychologically and consisting of emotional, cognitive and behavioral dimensions [28]. One of the main emotions related to aggressive behavior is anger, which plays an important role in eating disorders, and in particular, in BN [26, 29–31]. Indeed, some evidence suggests that anger may be a predisposing factor in the onset of binge eating in patients with BN [30, 32], with binge episodes serving as a means to lighten the experience of anger and the associated negative feelings [32].

The maintenance of anger has been associated with anger rumination, characterized by recurrent cognitions about frustrating experiences and recalling past anger experiences [33]. Anger rumination tends to exacerbate the existing anger effect and has been similarly associated with bulimic eating behaviors [34–36]. Finally, a tendency to aggressive and violent behavior has also been reported among adolescents and young adults with eating disorders, especially those with BN, in terms of acts of violence [37], the number of overtly expressed aggressive and violent behaviors [26], self-reported levels of covert and overt aggression [38] and relational aggression [39]. The vast majority of these studies, however, have been conducted with girls, which may be an important oversight, especially considering that the prevalence of bulimia problems tends to be underestimated in boys [13].

In addition, gender is one of the main predictors of differences in the forms of aggression [40]. Although it is generally agreed that boys use direct aggression significantly more often than girls [41, 42], findings on the use of social aggression have been mixed, with authors reporting either no gender differences [43–45] or that social aggression may be more pronounced in girls in late adolescence [46]. It has also been reported previously that manifestations of BN and associated comorbid disorders like substance abuse and affective problems in adolescents may be impacted by age and socio-economic status (SES) [47–49], with their prevalence increasing with age [50] and being higher in low-income families [51, 52], while both of these factors may also impact on aggression [53, 54]. Hence, both age and SES need to be taken into account when considering BN symptoms and their association with aggression.

In short, research suggests that problems with anger and aggression in adolescents with BN may lead to the manifestation of the disorder being increasingly complex, difficulties in treatment and a more complicated prognosis [23]. To the best of our knowledge, as yet, there has been limited research investigating the associations between BN and different dimensions of aggression among adolescents. Moreover, previous studies have been mostly conducted among clinical groups of adolescents and there is a lack of research on these associations in the general adolescent population, especially using a gender perspective. Finally, as aggression is a multidimensional concept taking different facets of aggression into account may be important when examining BN from both a clinical and theoretical perspective [25].

Importantly, BN research has often focused on the full presentation of the disorder based on a DSM-based diagnosis [6], while use of subthreshold diagnostic criteria has been less common [55]. Considering that the levels of comorbid mental health/medical problems and associated impairment among adolescents with subthreshold diagnostic criteria for eating disorders (EDs) may be high [10, 11], gaining a better insight into this phenomenon in individuals with clinically significant symptom levels, who may not necessarily seek help is an important undertaking.

Therefore, the goal of this study was to explore the association between a clinical level of bulimia symptoms (CLBS) and different dimensions of aggression in adolescents from the general population, focusing on gender, while controlling for age and SES. We hypothesize that bulimic symptoms will be more prevalent among girls than boys but that boys in the CLBS group will have higher anger and aggression scores than their female counterparts in this group.

## Methods

### Procedure

The data used in this study came from a school survey on a representative sample of students in the sixth to tenth grades (age 13–17) in the northwestern Russian city of Arkhangelsk, which has a population of around 349,000 and approximately 30,000 adolescents in the 13–17-year-old age range. The SES of the population is generally in line with that in other regions of Russia.

The study was approved by the ethics committee at the Northern State Medical University in Arkhangelsk (Russia). Subjects were selected from within general public schools that had been randomly selected from a list of all schools in each of the city’s districts, and from within classes that had been randomly selected from within each school. All of the students within each selected class were invited to participate in the survey. Students completed the survey questionnaire by themselves in their classrooms in the presence of their teachers during a regular school day. Parents were informed of the study and had the right to refuse their children’s participation. Children provided informed consent and had the right to refuse to continue participating at any point during the study. All information was collected anonymously (i.e., after they tore off a signed informed consent page, there was no means of identifying any individual who completed the survey). Those children who refused participation were provided with alternative tasks at school. A total of 3.6% of the students or their parents refused participation.

### Participants

The total sample consisted of 2847 participants but due to missing values on any variables of interest, 234 students were excluded from the analysis. The excluded group did not differ on any variables of interest apart from having a lower level of anxiety (*M* (*SD*s) = 12.11 (5.60) vs. 13.39 (5.67), *t* = 3.40, *p* < .001). The final sample consisted of 2613 students.

Boys, as compared to girls, were slightly younger (14.79 (1.11) vs. 14.96 (1.13), t = 3.73, p < .001), but did not differ in terms of SES (1.05 (1.14) vs. 1.00 (1.20), t = 1.12, ns). Most of the participants (75.7%) came from two-parent families. According to the students’ reports, 93.0% of their fathers and 94.4% of mothers had completed the equivalent of a secondary school education or beyond.

### Measures

*Disordered eating behaviors* were assessed using a shortened version of the Eating Disorder Diagnostic Scale [56]. The scale includes 4 statements on the occurrence of anorexia and bulimia symptoms in the previous 3 months: “I worried a lot about how to stop gaining weight” (anorexia/bulimia), “I felt fat even when others told me I am too thin” (anorexia/bulimia), “I felt very upset about my overeating or weight gain” (anorexia/bulimia) and “I ate large amounts of food even when I didn’t feel hungry” (bulimia). Response options included: “Not true” (scored 0), “Somewhat true” (1), and “Certainly true” (2). Cronbach’s α for the scale was 0.78. In addition, two further questions were used to assess the frequency (per week) of compensatory behaviors to prevent weight gain, including (1) vomiting or the use of laxatives and (2) fasting (skipping at least 2 meals in a row) or engaging in excessive exercise. Answer options were provided on a 5-point scale ranging from “0 times” (scored 0) to “More than 10 times” (4).

In order to create a proxy variable for a CLBS, the response options “Somewhat true” and “Certainly true” were used for coding the DSM-5 criteria for bulimia [1]. Diagnostic criterion A (recurrent episodes of binge eating) was coded based on the item: “I ate large amounts of food, even when I didn’t feel hungry”. B and C criteria (recurrent inappropriate compensatory behaviors, such as the use of laxatives, vomiting and fasting or excessive exercise, intended to prevent weight gain at least once a week for 3 months) were assessed with two items: “About how many times per week have you made yourself vomit or used laxatives to prevent weight gain?” (at least once) OR “About how many times per week have you fasted (skipped at least 2 meals in a row) or engaged in excessive exercise to prevent weight gain”? (at least once). Criterion D (body shape and weight unduly influence self-evaluation) was coded based on a positive response for either of the following statements: “I felt fat even when others told me I am too thin”, OR “I felt very upset about my overeating or weight gain”. Positive symptom scores for all four diagnostic criteria were used to create a binary variable (0/1), which was used in all further analyses and denoted as a CLBS. The present approach of identifying adolescents with clinically significant BN symptoms has been used previously with adolescent populations [15, 57, 58].

*The* *Trait Anger Scale of the State Trait Anger Expression Inventory (STAXI)* [59, 60] consists of 10 items that assess an individual’s general tendency to experience anger, either in the absence of a direct provocation or as a result of specific triggers such as criticism or unfair treatment by others (e.g. “I am a hot-headed person”, “I feel infuriated when I do a good job and get a poor evaluation” and “I get angry when I’m slowed down by other’s mistakes”). Students rated each item using a 4-point scale, ranging from almost never (1) to almost always (4). The scale has been validated previously in the Russian population and has demonstrated excellent internal consistency [60]. Cronbach’s α for the scale was 0.92 in the current study.

*The* *Anger Rumination Scale (ARS)* [33] measures the tendency to focus attention on angry moods, recall past anger experiences, and think about the causes and consequences of anger episodes. The Russian version of the ARS is comprised of 17 items (e.g. “After an argument is over, I keep fighting with this person in my imagination”, “When someone makes me angry I can’t stop thinking about how to get back at them”, and “I think about the reasons people treat me badly”). Participants were asked to report how well the items corresponded to their beliefs about themselves using a 4-point Likert-type scale where responses ranged from almost never (1) to almost always (4). The scale has been used with Russian adolescents previously [61]. In the present sample, Cronbach’s α for the scale was 0.95.

*Physical and verbal aggression* [62] were assessed with 5 items each (e.g. “Pushed or shoved somebody”, and “Punched someone in a fight” for physical aggression and “Teased others” and “Called others names” for verbal aggression). The students rated the frequency of their aggressive behaviors in the past 30 days on a 4-point scale (from “Never” (0) to “5 or more times” (3)). The total score for each of the scales could range from 0 to 15 with higher scores indicating more aggression. These scales have demonstrated good psychometric properties with Russian adolescents previously [61]. Cronbach’s alpha for the scales in the present study was 0.80 and 0.82, respectively.

*Social aggression* [62] was assessed with 9 items describing social/relational aggression towards a peer the respondent does not like (e.g. “Spread rumors/gossip”, “Told others not to be friends with him/her”, and “Told others bad things about this person”). The students were asked to respond to each item using a 4-point scale ranging from Almost never (1) to Almost always (4). The construct of social aggression is distinct from the measures of physical and verbal aggression and the scale has also previously shown good psychometric properties with Russian adolescents [61]. In the present sample, Cronbach’s α for the scale was 0.83.

Students’ information on family status (single-parent, 1/0), parental education (incomplete college education or lower, 1/0), and parental employment status (full time (0), part time (1) and unemployed (2)) was used to create a proxy variable for SES. A continuous variable was created where higher scores indicated having lower SES.

### Statistical analyses

SPSS version 25.0 was used to analyze the data. Chi-square and independent sample t-tests were performed when making univariate comparisons of demographic characteristics. General linear models (GLM) multivariate analysis of covariance (MANCOVA) was used to determine main and interaction effects across the fixed factors of a CLBS (1/0) (as described earlier) and gender (boys = 1, girls = 0), while adjusting for age and SES covariates.

MANCOVA analyses were conducted with anger and aggression scores (i.e. trait anger, anger rumination, social aggression, verbal aggression and physical aggression). Thus, we used a 2 (CLBS) X 2 (gender) design for assessing differences in anger and aggression. The unique contribution of each of the two fixed factors, the two covariates, and one interaction term were assessed through follow-up between-subject tests and unstandardized parameter estimates derived from the MANCOVA. Results are presented as means (*M*) and standard deviations (*SD*), and for individual outcomes in the between-subject tests as partial eta squared (η^2^), a common metric of effect size that represents the unique amount of variance explained by each predictor variable.

## Results

Participants in the study sample ranged in age from 13 to 17-years-old (M (SD) = 14.89 (1.13)). The composition of the sample was 59.5% female (N = 1554), an accurate reflection of the local public school population.

### CLBS and anger and aggression scores

A CLBS was significantly more prevalent in girls than in boys (13.4% vs. 3.5%, χ^2^ = 9.89, *p* < .001). When evaluating the differences in anger and aggression scores by a CLBS (see Table 1 for descriptive statistics (*M (SD*)) by gender and Table 2 for the tests of between-subjects effects), the main effect for the model was significant (Wilks’ lambda = 0.922; *F* (5, 2613) = 43.97, *p* < .001, η^2^ = 0.078). With regard to specific effects, the main effect for a CLBS was significant (Wilks’ lambda = 0.968; *F* (5, 2613) = 17.40, *p* < .000, η^2^ = 0.032), with higher levels of all anger and aggression scores in adolescents with a CLBS. The main effect for Gender was significant (Wilks’ lambda = 0.929; *F* (5, 2613) = 39.71, *p* < .001, η^2^ = 0.071), demonstrating higher levels of all anger and aggression scores among boys for all scales except for anger rumination (see Table 2).

**Table 1:** Anger and aggression scores (M (SD)) by bulimia symptoms in boys (B) and girls (G)

		CLBS	No CLBS
Trait anger	B	24.09 (6.47)	21.43 (6.88)
	G	21.98 (5.32)	19.88 (6.87)
Anger rumination	B	39.36 (12.60)	29.70 (10.30)
	G	35.60 (10.30)	31.77 (9.68)
Social aggression	B	18.50 (8.32)	14.80 (4.67)
	G	16.18 (4.77)	14.88 (4.34)
Verbal aggression	B	7.24 (4.68)	4.77 (3.94)
	G	4.68 (3.17)	3.69 (3.03)
Physical aggression	B	6.83 (5.02)	4.22 (3.78)
	G	2.40 (2.67)	1.98 (2.59)

M, Mean

SD, Standard Deviation

CLBS, Clinical Level of Bulimia Symptoms

**Table 2:** Effect sizes for each dependent variable (anger and aggression scores) (η^2^, p)

	Trait anger	Anger rumination	Social aggression	Verbal aggression	Physical aggression
Age	0.002, < 0.05	0.003, < 0.01	0.002, < 0.05	0.000, ns	0.003, < 0.01
SES	0.000, ns	0.001, ns	0.001, ns	0.000, ns	0.001, ns
Gender	0.003, < 0.01	0.000, ns	0.003, < 0.01	0.014, < 0.001	0.051, < 0.001
CLBS	0.006, < 0.001	0.022, < 0.001	0.014, < 0.001	0.012, < 0.001	0.011, < 0.001
CLBS x Gender	0.000, ns	0.004, < 0.01	0.003, < 0.01	0.002, < 0.05	0.006, < 0.001

η^2^, Eta squared

p, Significance value

SES, Socio - economic status

CLBS, Clinical Level of Bulimia Symptoms

ns, Non-significant

The main effect for SES was not significant (Wilks’ lambda = 0.997; *F* (5, 2613) = 1.77, ns, η^2^ = 0.003), suggesting a lack of difference in anger and aggression in relation to SES in the study group. The main effect for Age was significant (Wilks’ lambda = 0.985; *F* (5, 2613) = 8.04, p < .001, η^2^ = 0.015), indicating higher anger and aggression scores with increasing age. As concerns the interaction term, the effect for a CLBS x Gender was also significant (Wilks’ lambda = 0.987; *F* (5, 2613) = 7.05, p < .001, η^2^ = 0.013), suggesting that the differences in anger and aggression scores in relation to a CLBS were gender-specific, with the follow-up tests demonstrating differences in anger rumination, social aggression, verbal aggression and physical aggression, in terms of boys reporting higher scores in relation to a CLBS than girls.

As outcome differences might have been obscured by use of the MANCOVA analysis (i.e. by simultaneously assessing several outcomes in one model), in an additional analysis using uni-ANCOVA, each outcome was examined separately in order to determine whether the results that were obtained from the MANCOVA were the same for each individual risk factor. The results obtained were largely the same as those from the MANCOVA analysis.

## Discussion

This study examined the associations between a CLBS and anger and aggression in adolescents from the general urban population. A CLBS was more prevalent in girls than in boys (13.4% vs. 3.5%). This is much higher when compared to the earlier reported BN prevalence rates among adolescents where stricter diagnostic criteria were used [5–8]. Previous research among US inner-city youth [15] indicated that the use of less stringent symptom ratings, even with the same cut-off levels for compensatory behaviors, can produce an almost three-fold increase in the estimated prevalence of probable BN, which may explain the large differences in the estimates of BN prevalence reported in previous studies and emphasizes the importance of assessing subthreshold BN symptoms. Indeed, several studies have pointed to substantial similarities between subthreshold and full-threshold EDs, particularly between BN and subthreshold BN, in terms of the level of impairment, comorbidities, clinical presentation and medical complications [11, 55, 63, 64], suggesting that subthreshold ED symptoms may warrant closer attention, and may be potentially useful to consider in early prevention and intervention efforts.

Similar to earlier studies in adolescents diagnosed with BN [24, 25], we found a stronger association with anger and aggression in both genders in the CLBS group, compared to adolescents without a CLBS. Anger has been previously found to be associated with EDs in both clinical and community samples [25, 36]. However, some previous research has questioned this association given the possible confounding effect of comorbid psychopathology, such as anxiety and depression [65]. This being said, a recent study by Wakeford et al. [36] has provided further support for this association by showing that individuals who engaged in binge eating had higher levels of both anger and anger rumination even after comorbid anxiety and depression symptoms were controlled for.

While previous studies have largely focused on BN symptoms in girls, it has been suggested that the prevalence of BN symptoms in boys may be underestimated [13] and that the gender difference in the prevalence of BN symptoms may be much less pronounced [15]. Although we found that the prevalence of a CLBS was significantly higher in girls than boys, the present study not only supports the proposition of a lower gender ratio in BN (close to 1:4 in our study), but also provides some evidence that the associations between BN and different dimensions of aggression may also be present in boys, which in turn suggests both potential similarities in the mechanisms underlying the association (e.g. negative emotionality and impulsivity), while at the same time having possible implications for potential interventions, which may need to put an even heavier emphasis on issues of aggression in boys with BN symptoms.

Our findings showed that while boys scored higher than girls on verbal and physical aggression in both groups (and particularly within the CLBS group), gender differences in social aggression were minimal in the Non-CLBS group. This finding is in line with previous studies on gender differences in direct and indirect aggression in youth from the general population [41, 42]. Similar findings were also obtained in a meta-analytic review by Card et al. [66] that examined gender differences in child and adolescent direct and indirect aggression, which found clear support for the contention that boys engage in higher levels of direct aggression, but that there is only a minor gender difference in terms of indirect/social aggression, with slightly higher levels observed in girls.

The differences in aggression scores that were observed in relation to a CLBS may be linked to gender. In particular, gender differences in verbal and physical aggression, anger rumination and social aggression, were much more pronounced in the CLBS group, with higher levels in boys than in girls. It is possible that the presence of aggressive behaviors may change the prognosis of EDs as well as complicate their management [23] and thus, that screening for aggressive behaviors may be considered an important aspect of intervention programs. Given the relevance of rumination to ED psychopathology [67], as well as the gender-specific pattern of this association found in our study, rumination-focused interventions may be of value in the treatment of BN among adolescents, especially when the focus is on boys.

In both the CLBS and Non-CLBS groups, there was a relationship between age and anger and aggression with higher scores being associated with increasing age, which contrasts with the findings from some previous studies [25]. However, in another study age was reported as being a variable that influences the expression and reporting of aggressive tendencies [68]. These conflicting findings suggest that future research is thus needed to further delineate this association.

Considering that aggression and anger are frequent in patients with BN and may have a significant impact on its clinical aspects, dynamics, outcomes and the effectiveness of treatment strategies [38], it is possible that they may be related to the disorder’s underlying psychopathological mechanisms. Indeed, previous research has indicated that adolescents with BN often have problems with emotional regulation, negative affectivity, and impulse control [16, 17]. Similarly, some authors have commented on the presence of anger and anger rumination in behaviors with reduced self-control capacity, such as binge eating and impulsive drinking among college and university students [36, 69].

In addition, several studies [70, 71] have investigated another aspect of the association between aggression and eating problems, emphasizing the possible role of bullying among individuals with EDs. More specifically, being bullied is often associated with emotional problems [72], which could potentially contribute to the development of EDs [73]. Bullying is common during adolescence and coincides with maturation. As ED symptoms also often first appear during adolescence, problems in social relationships as a consequence of bullying may be crucial to the understanding of EDs [74]. These things need to be kept in mind and future research should disentangle the relationships between one’s own and others’ aggression in relation to EDs.

Hence, taking the potential underlying mechanisms of BN into account when planning interventions may be especially necessary, as research has emphasized the importance of addressing the issue of comorbidity in BN [75], and has indicated that aggressive manifestations in eating disorders may depend on the stage of the disease, and negatively influence on the course of therapy [76, 77].

The findings from the current study should be considered in the context of the study’s limitations. First, the cross-sectional design precludes causal inferences being drawn, while a longitudinal design would have helped us to determine the directionality of the observed associations. Second, the study sample consisted of a community sample of adolescents from northwestern Russia and caution is therefore required about generalizing these results to other populations, including clinical groups. Third, self-assessments of anger, aggression and bulimia symptoms were used in our study, which could have resulted in response bias. Being able to make use of other forms of data including that obtained through direct observation, and parent and teacher reports would have improved confidence in the findings. Finally, as several of the instruments used in this study have not been previously validated in Russia our findings should be considered with caution.

## Conclusions

This study adds to the small but growing body of evidence that there are gender-specific associations between anger, aggression, and bulimia symptoms among adolescents. The presence of anger and aggressive behaviors may influence the development of bulimia and considerably influence its prognosis leading to a more difficult course of treatment. Aggression may serve as a marker in individuals with eating disturbance who are unlikely to seek help resulting in a greater probability of not getting adequate treatment [26]. Our findings suggest that aggression and anger rumination are risk factors in bulimia and that clinicians should screen for these behaviors in children with BN as their detection may be important in ensuring the provision of effective treatments. In relation to aggressive behavior such treatment should include the regulation of anger and aggression in EDs. This approach is used for example, in the Brief Body and Movement Oriented Intervention (for more details see Boerhout et al. [78]) where one of the main ideas underlying the intervention is that there should be a focus on anger inhibition as an integral part of the ED and not merely as an isolated aspect of it.

## Data Availability

The data used in this study cannot be shared publicly due to the initial decision of the local ethical committee, as well as the restrictions included in the informed consent statement (where it was stated that the data would only be used by the research group and would not be transferred elsewhere). Requests to access the dataset should be directed to Vladislav Ruchkin.

## Abbreviations

CLBS: clinical level of bulimia symptoms
BN: bulimia nervosa
SES: socio-economic status
ED: eating disorder
